# Molecular and Cellular Mechanisms Underlying Neurologic Manifestations of Mosquito-Borne Flavivirus Infections

**DOI:** 10.3390/v15112200

**Published:** 2023-10-31

**Authors:** Britanie M. Blackhurst, Kristen E. Funk

**Affiliations:** Department of Biological Sciences, University of North Carolina at Charlotte, Charlotte, NC 28223, USA

**Keywords:** apoptosis, dengue virus, DNA damage, flavivirus, Japanese encephalitis virus, microglia, neurons, St. Louis encephalitis virus, West Nile virus, Zika virus

## Abstract

Flaviviruses are a family of enveloped viruses with a positive-sense RNA genome, transmitted by arthropod vectors. These viruses are known for their broad cellular tropism leading to infection of multiple body systems, which can include the central nervous system. Neurologic effects of flavivirus infection can arise during both acute and post-acute infectious periods; however, the molecular and cellular mechanisms underlying post-acute sequelae are not fully understood. Here, we review recent studies that have examined molecular and cellular mechanisms that may contribute to neurologic sequelae following infection with the West Nile virus, Japanese encephalitis virus, Zika virus, dengue virus, and St. Louis encephalitis virus. Neuronal death, either from direct infection or due to the resultant inflammatory response, is a common mechanism by which flavivirus infection can lead to neurologic impairment. Other types of cellular damage, such as oxidative stress and DNA damage, appear to be more specific to certain viruses. This article aims to highlight mechanisms of cellular damage that are common across several flavivirus members and mechanisms that are more unique to specific members. Our goal is to inspire further research to improve understanding of this area in the hope of identifying treatment options for flavivirus-associated neurologic changes.

## 1. Introduction

Flaviviruses are composed of positive-sense RNA consisting of three structural proteins (envelope protein, membrane precursor and capsid proteins) and seven nonstructural proteins (NS1, NS2a, NS2b, NS3, NS4a, NS4b, and NS5) [1]. Members of the flavivirus family include West Nile virus (WNV), Japanese encephalitis virus (JEV), Zika virus (ZIKV), dengue virus (DENV), St. Louis encephalitis virus (SLEV), yellow fever virus, tick-borne encephalitis virus, and Modoc virus [1]. Flaviviruses are known for having a wide tropism and have been shown to bind and enter different cell types using multiple different receptors, depending on cell type [2]. Once inside the cell, flaviviruses release the nucleocapsid-coated genomic contents into the cytosol and utilize the endoplasmic reticulum (ER) to replicate the viral genome and the Golgi apparatus to package and release all needed viral components to create new virion progeny [3,4,5]. Proteins within the genome assist in localization and development of virion components (reviewed in [4]). During uptake and replication, flavivirus components are recognized by receptors that lead to immune system responses [2]. The cell types infected and the immune response that is developed depends on the type of flavivirus and variant [6,7,8].

**Figure 1:**
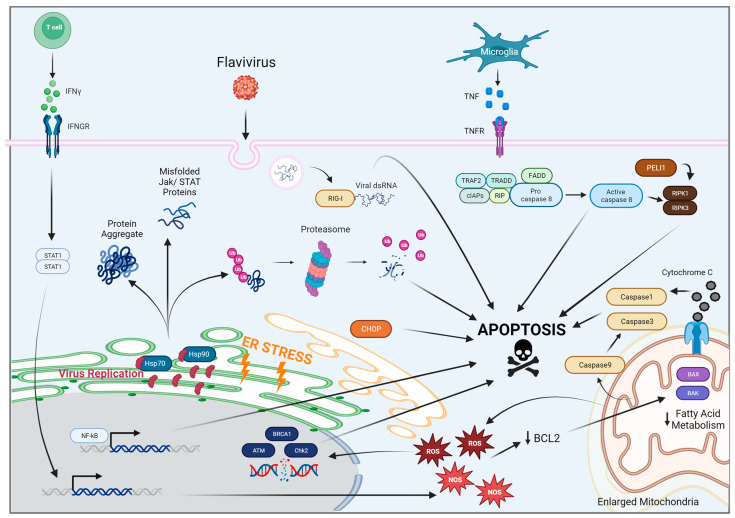
Mechanisms of apoptosis induced by flavivirus infections.

Recently, clinical research has connected viral infections with the development of neurodegenerative diseases later in life [9,10]. Understanding the connection between viral infections and the development of neurologic disease is critical to identifying treatments for predicting, preventing and treating diseases such as Parkinson’s disease, Amyotrophic lateral sclerosis, Alzheimer’s disease, and other forms of dementia. Studies have shown neurologic manifestations even in the absence of viral entry into the CNS, suggesting that these effects may be due to not only damage from viral infection directly but also from host-derived immune responses [11]. Apoptosis is a common response to viral infection, but each virus may initiate apoptosis via different mechanisms (Figure 1). Here, we review the current literature elucidating the molecular and cellular mechanisms through which flavivirus infection may cause neurologic sequelae during acute and post-acute infection periods. For this we have focused on family members with the greatest neurologic involvement, specifically WNV, JEV, ZIKV, DENV, and SLEV.

## 2. Flaviviruses and Associated Mechanisms of Neural Dysfunction

### 2.1. West Nile Virus

WNV is a mosquito-borne flavivirus that first appeared in North America in 1999 [12]. While the mortality rate of patients afflicted with WNV is 13%, it is noteworthy that WNV often culminates in neurologic sequelae affecting up to 50% of patients [13,14]. These neurologic manifestations of acute infection include meningitis, encephalitis, and acute flaccid paralysis. Neurologic sequelae during the post-infectious period are less well understood, with most studies comprising small cohorts of infected individuals. These studies indicate that older age and a diagnosis of West Nile encephalitis (WNE) rather than a less severe West Nile fever (WNF) are associated with persistent neurologic abnormalities [11,15]. This is postulated to arise from viral encephalitis resulting from direct penetration through the blood–brain barrier (BBB), coupled with inflammation provoked by the peripheral immune response [16]. Once across the BBB, WNV preferentially infects neurons, with a broad regional tropism preferentially affecting gray matter in the cortex, thalamus, brainstem, and spinal cord [17,18,19,20]. Studies have also demonstrated the capacity of WNV to infect glial cells, in particular astrocytes and microglia, but these have been in vitro rather than in vivo [21,22].

Neurologic dysfunction following WNV infection is likely due to, in part, neuronal and glial cell death. WNV can directly infect cortical neurons, spinal cord neurons and nerve root cells, causing subsequent cell death. This has been demonstrated through microarray of mice and in vitro stem cells infected with WNV, demonstrating increased presence of ubiquitinated Peli1, a protein involved in apoptosis and necroptosis [14,23]. Apoptosis, a less inflammatory form of cell death, appears to be the central mechanism of WNV-caused neuronal death [14].

Apoptosis can occur from mitochondrial damage, such as oxidative damage that results from many viral infections including WNV [24]. Genes associated with oxidative damage, such as iNOS and HO1 were transcriptionally upregulated during WNV infection in peripheral blood mononuclear cells [25]. Additionally, reactive oxidative species and increasing amounts of oxidative damage were correlated with increased risk of developing neurologic perturbations [26,27,28]. This form of apoptosis was first demonstrated in Neuro 2a cells, a murine neuroblastoma cell line, which were infected in vitro with WNV and shown to transcriptionally increase BAX, an apoptosis regulator [29]. BAX-formed homodimer clusters are activated, and subsequently lead to MAC complex formation and cytochrome C release from the mitochondrion, inducing apoptosis [30,31]. This was further confirmed by other studies demonstrating that the WNV capsid promoted complex formation of HDM2 with P53 to initiate BAX-dependent apoptosis [32].

WNV can also stimulate apoptosis via caspases 3, 8, and 9 activation [33]. Using a glioma cell line, Kleinschmidt et al. showed that WNV replication activated the apoptosis initiator caspases 8 and 9 as well as the apoptosis effector caspase 3 [33]. This apoptosis-inducing caspase cascade can be activated via upstream TNF interaction with TRADD and FADD [34]. Considering that TNF is produced by activated immune cells such as microglia, apoptotic death may not require direct viral infection. Following neuronal apoptosis, some cells may be replaced by neural progenitor cells; however, WNV is also capable of causing stem cell exhaustion, demonstrated in vitro [35]. Altogether, neurons are particularly susceptible to apoptosis following WNV infection, which may contribute to the neurologic dysfunction that appears during the acute phase of infection.

Beyond apoptosis, there are several other intracellular mechanisms that may contribute to cognitive dysfunction, including dysregulated protein folding. Although the connection between WNV infection and neurodegenerative disease is not well established, a case report of a patient with a history of WNV encephalomyelitis and poliomyelitis-like paralysis showed neurofibrillary tangle formation, a neuropathology composed of aggregated Tau protein that is a hallmark of Alzheimer’s disease [36]. This aggregated protein response may be due to the ability of WNV proteins to interact with important protein folding machinery. During viral replication, WNV NS5 binds the host heat shock protein 90 (HSP90) in the endoplasmic reticulum. This interaction has a two-fold effect in both facilitating proper folding of viral proteins and also reducing HSP90 interactions with its protein clients, which includes Janus kinases (JAKs). This results in JAK instability, dysregulated JAK/STAT signaling and ultimately, uncontrolled WNV replication [32]. Additionally, the WNV capsid protein can interact with host HSP70 and negatively regulate its chaperone function [37]. In 293T cells this induced cell cycle arrest, but how this interaction may impact neurons, which are post-mitotic, is less clear. Both HSP70 and HSP90 are important regulators in protein homeostasis and the unfolded protein response (UPR) [38]. HSP70 and HSP90 are implicated in many neurodegenerative diseases, which are often characterized by the accumulation of pathological protein aggregates [39]. In human neuroblastoma cells and primary rat hippocampal neurons, WNV activated multiple UPR pathways, likely by hijacking endoplasmic reticulum-derived structures during viral replication [40]. WNV-infected neuronal cells accumulated ubiquitinated proteins in mice and in neuroblastoma cells [41], which was shown to be due to the inhibition of autophagy, a mechanism used by cells to degrade protein aggregates [42]. Autophagy is initiated by a pathway of proteins involving AMP-activated protein kinase (AMPK). In WNV-infected neuroblastoma cells, AMPK was ubiquitinated and degraded, thus reducing autophagy and increasing unfolded protein aggregates [42]. Intracranial infection with an attenuated strain of WNV that can replicate but does not induce protein aggregate accumulation showed reduced cytotoxicity and reduced cellular degeneration in infected brains [42].

A relatively common neurologic manifestation of WNV infection involves difficulty with movement, including tremor, neuromuscular weakness, poor balance, and decreased reflexes [43,44]. Many of these neurologic symptoms, including tremor, parkinsonism, postural and gait instability, and mobility impairment are associated with white matter pathologies that can be visualized using magnetic resonance imaging technologies [45,46,47,48,49,50]. Similarly, several case studies of patients infected with WNV exhibit white matter pathology in their brains and spinal cords [51,52]. There is little-to-no evidence to suggest that WNV infects oligodendrocytes and Schwann cells, which are responsible for myelinating neurons in the CNS and PNS, respectively. Rather, these symptoms may be caused by inflammatory responses in the white matter, as is the case in multiple sclerosis, a neurodegenerative disease with prominent motor and gait dysfunction that is believed to be triggered by the immune system [53].

The impact of immune cells on neurologic dysfunction is of particular interest. As suggested above, white matter pathology and demyelination are likely caused by inflammatory responses, rather than direct infection of myelinating cells. Additionally, persistent activation of the brain-resident immune cell, microglia, contributes to post-infectious cognitive dysfunction. Synapse elimination by microglia occurs during normal development and throughout adulthood to remove redundant or inefficient synapses [54]. However, inflammatory responses to WNV infection also result in synapse removal. Infecting mice with an attenuated strain of WNV that harbors a point mutation in NS5, WNV-NS5-E218A caused spatial learning deficits on the Barnes maze at 1 month after the virus had cleared. This cognitive dysfunction correlated with reduced synapses in the hippocampus of infected mice and the identification of synaptic proteins within phagosomes of microglia without significant loss of neurons [43]. A follow-up study showed that CD8^+^ T cells recruited to the brain during infection drove prolonged microglial activation and synapse elimination through IFN-γ signaling [55,56,57].

We have recently reviewed the literature discussing the role of antiviral CD8^+^ T cells as potential mediators of cognitive impairment [58]. As noted above, persistent activation of microglia by CD8^+^ T cells drove synapse elimination by microglia [55]; however, microglia and their ability to recruit antiviral CD8^+^ T cells are necessary for clearance and survival from WNV infection [59]. Expression of tumor necrosis factor-related apoptosis-inducing ligand (TRAIL), perforin, or Fas ligand by CD8^+^ T cells caused direct killing of infected neurons, which, although important for controlling WNV replication in the CNS, can contribute to neurologic dysfunction [60,61,62,63]. Similarly, IFN-γ, an important antiviral cytokine expressed by CD8^+^ T cells induced neurotoxicity by complexing the IFN-γ receptor with the AMPA glutamate receptor. Signaling through this complexed receptor via IFN-γ caused Ca^2+^ influx in glutamatergic neurons and, ultimately, neuronal excitotoxicity in the form of dendritic beads [64].

After the virus is cleared from the brain, some CD8^+^ T cells are retained as resident memory T cells [55]. The CXCL16/CXCR6 signaling axis has recently been identified as maintaining WNV-specific CD8^+^ T cells within the brains of post-infectious mice and as contributing to their transition to a resident memory phenotype [65]. In this context, CXCR6^+^CD8^+^ T cells in the brain caused persistent glial cell activation and synapse elimination. Another recent study found that CXCL16/CXCR6 signaling orchestrated the retention of resident memory T cells within the brains of human Alzheimer’s disease patients and mouse models of Alzheimer’s disease [66]. This study reported that CXCL16/CXCR6 crosstalk between microglia and T cells restricted Alzheimer’s disease pathology due to their immunosuppressant activity. Thus, whether T cells are helpful or harmful in disease is likely dependent on context and a balance of inflammatory and immunomodulatory activities [67].

Astrocytes are glial cells that are the most numerous cell types in the CNS. Their functions in the brain are wide-ranging and include supporting neurons forming the BBB and participating in innate immune responses [68]. During infection, astrocytes detect viral PAMPs and upregulate IFN-stimulated genes, which limit viral replication, and inflammatory cytokines and chemokines, which recruit professional immune cells such as brain-resident microglia and infiltrating lymphocytes to the site of infection [69]. In addition to their beneficial roles during infection, astrocytes contribute to long-term neurologic impairments post infection by disrupting BBB function and amplifying microglia and T cell inflammation (reviewed in [69]). Astrocytes control entry of WNV into the CNS, in part by regulating the permeability of the BBB. Mice with astrocyte-specific loss of the receptor for IFN-α and IFN-β exhibited increased mortality from WNV, due to increased permeability and greater viral entry into their hindbrains [70]. In fact, whether a specific strain of WNV is neuropathogenic may be due to whether it can productively infect astrocytes [71]. BBB and neurovascular disruption are common in neurodegenerative diseases [72], and likely contribute to the ability of peripheral immune cells to enter into the CNS and cause persistent damage, as described above. In addition to their role in the BBB, astrocytic expression of inflammatory cytokines can cause additional neural dysfunction. Recently, IL-1 expression by inflammatory astrocytes has been shown to limit adult neurogenesis by impairing neural progenitor cell homeostasis, resulting in impaired spatial learning [73]. Furthermore, IL-1R1 signaling in neural stem cells promotes their development into IL-1β-expressing astrocytes instead of newborn neurons, which further diminishes synapse recovery and spatial learning [35]. Altogether, astrocytes can be beneficial as support cells, but can also contribute to prolonged inflammation and cognitive dysfunction.

### 2.2. Japanese Encephalitis Virus

Like WNV, JEV is a flavivirus that is spread via mosquitoes. JEV is a leading cause of encephalitis (namely Japanese encephalitis, JE) in Asia and the Western Pacific, with rates of 30% fatality and 30–50% long-term neurologic or physical sequelae in survivors [67]. Approximately 75% of JE cases occur in children younger than 15 years of age, and although there is no cure for JE, there are vaccines available that demonstrate effectiveness when large-scale vaccination programs are implemented [74]. In patients with neurologic disease, JE progresses in three stages. The first “prodromal” stage is characterized by flu-like symptoms of general malaise, mild fever, muscle pain, vomiting and diarrhea. The “acute” second stage is marked by reduced consciousness, seizures, and parkinsonism, which may progress rapidly and become lethal. The “post-acute” third stage can be characterized by prolonged neurologic sequelae, including cognitive and language impairment, poliomyelitis-like flaccid paralysis, and parkinsonian syndrome [74,75]. This section will summarize the literature that discusses mechanisms which contribute to these neurologic manifestations post infection, as well as discussing long-term changes that may contribute to progressive neurologic disease later in life.

Once in the CNS, JEV is highly neurotropic [76,77,78], and, like WNV, causes neuronal apoptosis. This has been demonstrated via proteolytic cleavage of the BAX protein from the endogenous p21 to the proapoptotic p18 form, which induced cytochrome c release from the mitochondria and, ultimately, cell death [79]. Apoptosis may be initiated by activating the RIG-I signaling pathway, which is involved in sensing viral RNA in the cytoplasm. In BV2 cells, a mouse microglial cell line, JEV promoted mitochondrial-dependent apoptosis between 24 and 60 h of infection, a phenotype that was alleviated by interfering with RIG-I signaling [80]. In a mouse model of JEV infection, motor neurons similarly exhibited RIG-I-dependent apoptosis, which was blocked by silencing RIG-I expression with antisense RNA oligonucleotide treatment [81]. Additionally, JEV promoted apoptosis by reducing expression of anti-apoptotic proteins Bcl-6 and p21 via the downregulation of STAT3 and the FoxO transcription factor [82].

Like WNV, JEV can cause ER stress and UPR; however, whereas this initiated the accumulation of ubiquitinated proteins in WNV-infected neuronal cells due to dysregulated autophagy [41], in JEV this resulted in apoptosis in JEV-infected neuronal cells and potentially aggregation of proteins from Sequestosome1 production [83,84]. JEV replicates in the cytoplasm but uses the cell’s secretory pathways to release mature virions, causing proliferation and hypertrophy of the rough ER where virus particles accumulate [85]. JEV NS 4B triggered the UPR by inducing dimerization and activation of the R-like ER kinase (PERK), the activation of which led to phosphorylation and activation of the cell death-related transcription factor CHOP [84,86]. Thus, apoptosis of JEV-infected neurons is likely a major culprit of neurologic complications from JEV infection; however, other molecular and cellular mechanisms may also contribute to neurologic sequelae.

Fatty acid metabolism typically occurs in the mitochondria and increases during flaviviral infections to allow for efficient viral replication. However, during JEV infection, the viral NS5 interacted with the mitochondrial trifunctional protein, impairing fatty acid β-oxidation and reducing fatty acid metabolism [87]. Reduced fatty acid oxidation resulted in increased free fatty acids, which induced expression of proinflammatory cytokines and neuroinflammation [88]. Despite decreased fatty acid oxidation, increased oxidation from NOS and ROS can cause cognitive dysfunction via metabolic dysregulation from mitochondrial damage, DNA damage and neuronal apoptosis, causing prolonged inflammatory responses [28,89,90].

As noted above, neurons are the primary targets of JEV, but glial cells also contribute to neurologic complications of JEV infection. As the brain-resident immune cell, human microglia exposed to either live or inactivated JEV in vitro upregulated the expression of inflammatory chemokines CCL2, CXCL9, and CXCL10, which are involved in immune cell recruitment to the brain [91]. Microglia also demonstrated mitochondrial dysfunction that resulted in increased inflammation. In cultured rat glial cells, JEV caused oxidative damage after 24 h of infection, as measured by the amount of nitric oxide in the media [92]. The role of astrocytes in the neuroimmune response is also becoming better understood. In a human astrocyte cell line, JEV caused expression of inflammatory cytokines TNF, IL-6, IL-1β, CCL-5, and IFN-β [93]. This was due to enhanced phosphorylation of p21-activated kinase 4 (PAK4), which promoted MAPK signaling via NF-kB and phosphorylation of AP-1. It is unknown whether this was due to productive JEV infection in astrocytes or whether in vivo astrocytes are infected. In a nonhuman primate model of intranasal inoculation, JEV primarily targeted neurons in the thalamus and brain stem, and a few JEV-positive microglia were identified, but there was no evidence of JEV-positive astrocytes. Even in the absence of direct infection, investigators reported astrocyte activation and astrogliosis, along with expression of TNF, IFN-α, iNOS, NT, and MMP-2 [94]. Myint et al. suggested that astrocytes may contribute to bystander neuronal apoptotic cell death through their inflammatory effects [94]. To better understand the secondary wave of apoptotic neuronal cell death, Swarup et al. tested whether silencing the TNF receptor-associated death domain (TRADD) with small interfering RNA (siRNA) reduced neuronal apoptosis and subsequent glial activation. Their results showed that TRADD mediated JEV-induced neuronal death, and its silencing alleviated the resultant neuroinflammatory response, including microglial and astrocyte activation as well as CNS-infiltrating leukocytes [95].

Unlike viruses with DNA genomes, it is widely believed that RNA viruses cause acute infections that are rapidly cleared from the host; however, there is some evidence to suggest that viral RNA can persist following acute infection, especially in “immune-privileged” sites, such as the brain [96]. In many cases, it is likely that recovered viral RNA is non-infectious genome fragments, but there are some instances where persistent viral RNA can be cultured in permissive cells. In a microglial cell line, cells remained productively infected with JEV, capable of releasing virions that were infectious to mouse neuroblastoma cells for up to 16 weeks [97]. In another culture system, primary human microglia and human blood monocyte-derived microglia were able to transmit JEV to neighboring cells in a contact-dependent fashion [91]. Interestingly, in both of these systems, JEV did not induce cytopathic effects in infected microglia, introducing the possibility that microglia may be capable of acting as a viral reservoir for JEV, potentially sustaining brain pathogenesis or prolonging associated neuroinflammation.

As noted above, acute flaccid paralysis and parkinsonian syndrome are neurologic complications of JEV. Tseng et al. noted that mice inoculated intravenously with JEV exhibited symptoms of infection including hindlimb paralysis, which was associated with severe inflammation and axon demyelination in the brain. This was further evidenced by the presence of antibodies specific to myelin basic protein (MBP) in their sera and MBP-specific T cells in their spleens, similar to what is often seen in models of multiple sclerosis [98]. JEV has also been associated with Guillain–Barré syndrome, which primarily affects the peripheral nerves [99]. To study the effects of JEV on peripheral nerve injury, Yang et al. developed a mouse model of infection that consistently developed peripheral motor deficits. These motor deficits were associated with sciatic nerve demyelination and axonal degeneration as well as reduced nerve conduction velocity, as measured by electromyography [100]. Viral loads in the injured sciatic nerve, inflammatory cell infiltration, and inflammatory cytokine expression were all positively correlated with sciatic nerve injury, suggesting that JEV-induced peripheral nerve injury is likely due both to the virus directly and to inflammation associated with the anti-viral response [100].

### 2.3. Zika Virus

ZIKV is a mosquito-borne flavivirus that was first isolated in 1947 and became of broad interest to the medical community in 2013–2017, when the virus emerged in the Americas [101]. This ZIKV outbreak was of particular concern to pregnant women due to the increased risk of fetal demise and microcephaly [102], but has also been associated with Guillain–Barré syndrome [103] and ocular complications [104]. Unlike WNV and JEV, ZIKV can infect a broad range of cell types in the brain including neurons [105], astrocytes [105,106], oligodendrocytes [106], Schwann cells [107], neural progenitor cells [108], glial progenitor cells [109], and pericytes in the choroid plexus [110]. ZIKV has been shown to target the ventricular and subventricular zones of the brain in fetuses, and is a primary cause of resultant microcephaly [111,112,113].

Microcephaly is a birth defect that is characterized by a significant reduction in brain size and intellectual ability. It is thought to be caused by the combined effects of excessive death of mature brain cells and impaired proliferation of neural progenitor cells [111]. ZIKV infection promoted apoptosis of neurons, astrocytes, oligodendrocytes, microglia, and neural progenitor cells [106,108,109,114,115]. Pathways activated by ZIKV to induce apoptosis were recently reviewed by Turpin et al. [116]. Of particular significance is the death of neural progenitor cells, which has been shown to cause microcephaly in mouse and nonhuman primate models of fetal infection [108,117]. In addition to abolishing the neural progenitor cell population, ZIKV infection dysregulated the Notch pathway during human neural stem cell differentiation, promoting their development into astrocyte progenitors instead of neuronal and oligodendrocyte progenitors [118,119]. ZIKV can also infect and promote cell death in neural progenitor cells in immunodeficient adult mouse models, leading to reduced adult neurogenesis [120].

While apoptosis can initiate an immune response due to phagocytosis of apoptotic bodies and the subsequent antigen presentation, necroptosis and pyroptosis are lytic forms of cell death that stimulate more robust inflammatory responses [121]; however, necroptosis and pyroptosis serve different cellular purposes. Whereas pyroptosis is a primary cellular response to initiate an inflammatory reaction, necroptosis is initiated as a backup form of cell death when apoptosis is blocked, such as during infection [122,123]. In a neonatal mouse model, ZIKV infection caused pyroptosis of neural cells including neural progenitor cells, astrocytes, and microglia via the activation of caspase-1 and gasdermin D [124]. Necroptosis is orchestrated by RIPK1 and/or RIPK3, which activate the executioner MLKL. In human astrocytes, ZIKV infection led to necroptotic cell death and the release of proinflammatory cytokines IL-6, IL-8, and IFN-β [125]. In this system, necroptosis was suggested to be protective, as inhibiting RIPK3 increased viral replication. However, Daniels et al. showed that RIPK3 signaling restricted viral replication via neuroinflammatory responses, independently of cell death, in WNV-infected mice and altered neuronal metabolism to suppress viral replication in ZIKV-infected mice [125,126]. Altogether, cell death of neural progenitor cells via apoptosis, necroptosis, and pyroptosis leads to reduced brain volume and microcephaly, and ZIKV infection leads to an inflammatory brain environment.

Most people infected with ZIKV do not develop any symptoms. For those who do, symptoms generally start 3–14 days after exposure and resolve after 2–7 days. Virus is cleared from the serum 3–16 days after symptom onset [127]; however, viral RNA may be able to persist in immune-privileged sites, such as the brain, placenta and testis [128,129,130]. In a mouse model in which neonates were infected at postnatal day 1, most animals survived the acute infection and seemingly recovered; however, brain parenchyma of convalescent mice showed persistent viral antigen, apoptotic neurons, activated microglia and astrocytes, and cellular infiltrates even 1 year post infection. Furthermore, these convalescent mice exhibited long-term motor and behavior defects at 1 year post infection, but it was not clear whether these defects were due to the replicating virus specifically or to the collateral persistent neuroinflammation [129]. Research by Garber et al. showed that IFN-γ production by infiltrating antiviral T cells promoted neuronal apoptosis as well as microglia-mediated synapse elimination following ZIKV infection in adult mice, and contributed to spatial learning deficits [55]. This IFN-γ-mediated apoptosis may be due to synergistic effects of TNF, which promotes nitric oxide production and mitochondrial-mediated cytochrome c release, as has been shown in mesenchymal stem cells [131]. Another study showed that ZIKV caused synapse elimination that resulted in memory impairment in mice; a phenotype that could be rescued by neutralizing TNF signaling or by blocking either microglial activation or complement cascade signals [132]. Together, these data indicate that neuroinflammation may underlie some neurologic deficits associated with ZIKV infection, and point to neuroinflammation as a potential therapeutic target.

Much of the research into neurologic complications of ZIKV infection has focused on the severe congenital deformation, microcephaly; however, a proportion of individuals infected with ZIKV develop neurologic complications that may be caused, in part, by demyelinating effects of the virus. In a prospective observational cohort study of hospitalized adults with new-onset acute parainfectious or neuroinflammatory disease in Brazil in early 2016, 88% of patients had molecular or serological evidence of recent ZIKV infection. Of the 35 cohort patients with ZIKV infection, 18 had Guillain–Barré syndrome, 5 had encephalitis, 2 had transverse myelitis, and 1 had chronic inflammatory demyelinating polyneuropathy [133]. In a neuroimaging study, structural and functional magnetic resonance imaging of adult patients in the subacute phase of ZIKV infection showed nerve velocity conductivity values consistent with an acute demyelinating inflammatory polyneuroradiculopathy [134]. The negative effect of ZIKV on myelinating cells has been shown in animal and cell culture models. Several studies have shown that oligodendrocytes are susceptible to ZIKV infection, which resulted in their cell death both in vitro and in vivo [106,135]. Interestingly, while Guillain–Barré syndrome manifests in the PNS, CNS oligodendrocytes are more susceptible to damage from ZIKV than PNS Schwann cells [136].

Unique to ZIKV, compared to other flaviviruses, is the robust DNA damage that occurs during infection. Using RNA-seq analysis, investigators found that ZIKV-infected neuronal cells down-regulated genes involved in DNA repair, highlighting pathways involved in cell-cycle-checkpoint control, ATM signaling, and BRCA1 DNA damage response [137]. Several cell culture models support the hypothesis that ZIKV infection can cause cell cycle arrest that leads to DNA damage. In iPSC-derived human neural progenitor cells, ZIKV increased total protein levels of the tumor suppressor gene p53 and also induced its phosphorylation at Ser15, which resulted in genotoxic stress and apoptosis [138]. In neural progenitor cells, ZIKV hijacked a critical DNA damage repair enzyme, polynucleotide 5′-kinase 3′-phosphatase (PNKP), sequestering it in the cytoplasm during viral replication, rendering the cell unable to repair damaged DNA [139]. In another cell culture model of human neural progenitor cells, ZIKV halted replicating cells during S phase, inducing DNA damage. While ZIKV activated the ATM/Chk2 checkpoint, it prevented activation of the ATR/Chk1 checkpoint pathway [140]. In iPSC-derived astrocytes, ZIKV caused mitochondrial dysfunction and ROS release, inducing DNA breakage and activation of DNA damage response pathways [141]. All together, these studies indicate that ZIKV-infected cells acquire DNA damage that the cell is unable to repair, leading to dysfunction and cell death. Interestingly, mutations in several DNA repair pathway proteins, including PNKP and p53, can cause genetic microcephaly syndromes, further supporting the evidence that DNA damage and repair can underlie microcephaly [142].

### 2.4. Dengue Virus

DENV is the most common flavivirus, causing 100 million infections per year [143]. The cases of DENV have been increasing on average every year since the first case in 1986 [144]. DENV outbreaks have occurred in six out of seven continents, posing a large threat to the greater population as cases increase. Even with a low mortality rate, 20,000 people die yearly from DENV, with the greatest risk to the elderly population who suffer from severe illness and long-term disease [144]. Several vaccination strategies have been tested, but only two have been approved for public use: Dengvaxia by the FDA and Takeda in the European Union, the United Kingdom, Brazil, Argentina, Indonesia, and Thailand [145]. DENV vaccines have been plagued by controversy because of their propensity to enhance the entry of the virus into target cells and facilitate its replication, rather than neutralizing them, a phenomenon known as antibody-dependent enhancement [146]. Although not traditionally considered a neurotropic virus, DENV has recently been shown to be present in the cerebrospinal fluid and to cause damage to the BBB, suggesting CNS involvement [147]. Neurologic effects have been classified into those of the CNS and eyes, those associated with the PNS, and those occurring in the post-infectious convalescent stage, which are thought to be immune-mediated syndromes. The most common neurologic complication of DENV infection is encephalopathy, which is an impaired mental state or altered consciousness that is due to dysfunction of non-CNS body systems, such as liver failure and shock [148,149]. A less-common result of DENV infection is encephalitis, which is due to the direct brain infiltration by the virus [150]. Ocular manifestations include visual loss, pain, eye flashes and floaters, and photophobia. Other neurologic sequelae include stroke, immune-mediated neurologic syndromes, and neuromuscular complications [148]. Case reports have described rare incidences of DENV-associated demyelinating diseases, such as acute hemorrhagic leukoencephalopathy [151], neuromyelitis optica spectrum disorders [152], transverse myelitis [153], and Guillain–Barré syndrome [154]; however, these appear to be uncommon sequelae.

All flaviviruses are vector-borne viruses, frequently transmitted via mosquitos. Viral infection generally begins in the skin, and is then transmitted to the brain. The mechanism by which these viruses gain entry to the brain is not fully understood, but requires passage through the BBB in some way. It is hypothesized that some flaviviruses are able to reach the CNS without overt disruption to the BBB [155]; however, DENV is particularly known to cause hemorrhagic fever and stroke. DENV can infect mouse brain endothelial cells (mBECs) in vitro, causing changes in the cellular localization of tight junction proteins ZO-1 and Claudin-1. Infection of these cells and subsequent mislocalization of these proteins reduced the barrier function, causing decreased transendothelial resistance and increased permeability, as well as transcriptional upregulation of cellular adhesion molecules and immune mediators that increase immune cell transmigration [156]. DENV infection in human brain microvascular endothelial cells (hBMECs) activated the RIG-I pattern recognition receptor and increased production of IFN-α and IFN-β. This promoted higher levels of the cell adhesion molecule ICAM-1, which increased leukocyte recruitment and enhanced inflammatory responses [157]. A barrier to understanding DENV pathogenesis in vivo is the limited susceptibility of mice to most strains of the virus [155]. Most research studying the neurotropism of DENV have used direct intracranial inoculation, which bypasses the BBB. Using a strain of DENV that was neuro-adapted, Velandia-Romero et al. developed an intraperitoneal mouse model of infection that exhibited encephalitis, severe BBB damage, and plasma leakage [158], all of which are cardinal features of DENV hemorrhagic fever [159]. DENV also increases the short-term risk of stroke, both hemorrhagic and ischemic [160].

Ocular manifestations of DENV infection include subconjunctival hemorrhage, uveitis, and maculopathy, but the molecular and cellular mechanisms underlying these manifestations are not well studied. Retinal cells including pigment epithelial cells and endothelial cells, which comprise the blood–retinal barrier, can be infected by DENV [161]. Like cells in the BBB when infected, retinal endothelial cells initiated an antiviral immune response including expression of IFN-α and IFN-β and upregulated VCAM-1 for leukocyte recruitment. Infection of the epithelial cells also altered the expression of tight junction proteins and decreased transcellular impedance, consistent with increased barrier permeability [161]. The prevalence of retinopathy varies across different DENV outbreaks, with the disease specifically linked to the serotype 1 virus. To test whether the prevalence of retinopathy corresponds to the infectivity and response of infected cells to different strains of the virus, human retinal pigment epithelial cells were infected with six strains of DENV virus (all serotype 1). Results showed that strains of virus associated with a higher prevalence of retinopathy produced higher viral titers, more robust antiviral signaling, and greater barrier permeability than strains associated with lower retinopathy prevalence [162]. Together, these data indicate that an important cause of DENV-associated neurologic sequelae may be through infecting epithelial cells and compromising the integrity of the blood–brain and blood–retinal barriers.

In fatal cases of infection, DENV has been identified in neurons, brain endothelial cells, oligodendrocytes, and microglia [158,163]. Similar to previously mentioned flaviviruses, DENV causes neuronal cell death via apoptosis, necroptosis, and pyroptosis [164,165]. DENV caused apoptosis in vitro in immortalized cell lines such as Neuro 2a cells as well as in vivo in infected neonatal mice, evidenced by oligonucleosomal DNA fragmentation, a late-stage marker of apoptosis [166,167]. DENV neuronal damage was restricted to the hippocampal neurons, specifically the pyramidal layer of the choroid ammonia (CA) region [167]. These apoptotic effects may be due to both the direct action of arachidonic acid and superoxide anion on the mitochondrial membrane or indirectly from the products of NF-kB activation and apoptosis-related gene transcription [168]. In an attempt to directly compare cell death pathways among different flaviviruses, Jhan et al. infected neonatal mice with DENV, JEV, or ZIKV and performed transcriptomic RNA-seq analysis on brain samples at 5 days post infection. Results showed similar gene expression patterns, with all three viruses showing patterns consistent with apoptosis, pyroptosis, and necroptosis, but to slightly different degrees [165].

With its impact on the BBB, DENV can cause severe inflammation in the brains of infected individuals. RNA-seq analysis of DENV-infected neonatal mice showed that both brain-resident microglia and infiltrating macrophages upregulated inflammatory genes [165], and in a mouse model of intracerebral infection, DENV-infected brains showed immunopathological effects including reactive gliosis, hypertrophied microglia, astrocytosis, and cellular infiltration [169]. This immune-related pathology replicates what is seen in human patients, in which patients with higher concentrations of inflammatory mediators including IL-1α, IFN-γ, IL-10, IL-8, and VCAM-1 in their plasma experience a more severe disease course. Moreover, patients with severe disease show slower resolution of those inflammatory signals [170]. However, whether the increased inflammation causes more severe disease or severe disease necessitates intense immune response cannot be distinguished from this study. Furthermore, although inflammatory microglia can exert damaging effects by engulfing neuronal synapses and recruiting potentially damaging lymphocytes, they are, in fact, necessary to facilitate the antiviral immune response and clear the virus. Pharmacologic depletion of microglia resulted in increased viral replication, neuropathy, and mortality in both DENV-infected and WNV-infected mice [59,170].

### 2.5. St. Louis Encephalitis Virus

Another mosquito-borne flavivirus that is less well studied is SLEV, a positive RNA genome virus with a broad geographic distribution ranging from Canada to Argentina. The annual number of reported cases fluctuates each year due to periodic epidemics, but the largest outbreak occurred in 1975 in the central United States, with 2000 reported cases [171]. Most cases are asymptomatic, and thus go unreported. Severe, neuroinvasive disease resulting in encephalitis is uncommon, but is more likely in the elderly population in which an infected individual’s likelihood of succumbing to infection increases [172]. Although adult survivors may have prolonged periods of illness that may involve neuropsychological illness [173] and motor function [172], pediatric patients under the age of 10 were reported to have a higher incidence of neurologic sequelae, including convulsion and intellectual disability, but showed considerable improvement with time [174].

Experimental systems for studying SLEV are quite limited, but have been developed in cell culture and in mice. In a human mononuclear cell line and in Neuro 2A cells, SLEV caused apoptotic cell death via transcriptional upregulation of pro-apoptotic BAX [175]. In mice infected intracranially with SLEV, mortality occurred at seven days post infection and tissue damage in the brain was evident. SLEV targeted neurons and glia for infection and replication, causing neuronal death and cytokine production, which then recruited lymphocytes and macrophages [176]. One study demonstrated that astrocyte infection led to increased neuroinvasion and astrocyte death, evidenced by caspase 3 activation [177]. Overall, this model recapitulated several pathologies evident in human SLEV patients, which may be useful in future efforts to understand the molecular and cellular mechanisms underlying neurologic consequences of this virus.

## 3. Conclusions

**Figure 2:**
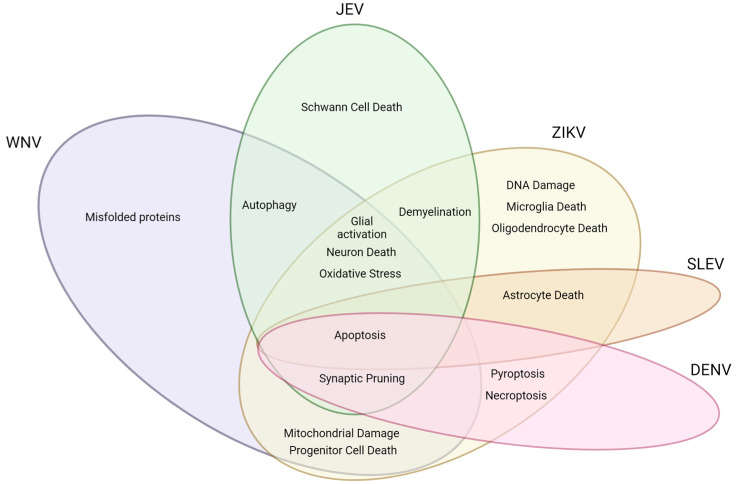
Common and unique molecular and cellular mechanisms driving neurologic dysfunction during acute and post-acute flavivirus infection.

Neurotropic flavivirus infections can lead to neurologic consequences both in the acute and post-acute phases, which can include paralysis in one or more limbs, Guillain–Barré-like syndrome, parkinsonism, cognitive dysfunction, and tremors. As illustrated in Figure 2, some of these mechanisms are related to common features seen in several neurotropic flaviviruses. For example, cell death from apoptosis, necroptosis, and/or pyroptosis is a common feature among the viruses discussed here. Inflammation caused by activation of brain-resident microglia and infiltrating lymphocytes is also common among neurotropic viral infections. Other clinical manifestations are more specific to certain viruses, such as the microcephaly and DNA damage caused by ZIKV. Knowledge of the common and unique mechanisms that lead to the development of these neurologic manifestations will provide an avenue for improved understanding of how viral infections can culminate in lifelong neurologic dysfunction and disability.
